# Trefoil Factor 3, Cholinesterase and Homocysteine: Potential Predictors for Parkinson’s Disease Dementia and Vascular Parkinsonism Dementia in Advanced Stage

**DOI:** 10.14336/AD.2017.0416

**Published:** 2018-02-01

**Authors:** Jing Zou, Zhigang Chen, Caiqian Liang, Yongmei Fu, Xiaobo Wei, Jianjun Lu, Mengqiu Pan, Yue Guo, Xinxue Liao, Huifang Xie, Duobin Wu, Min Li, Lihui Liang, Penghua Wang, Qing Wang

**Affiliations:** ^1^Department of Neurology, and; ^2^Department of Emergency, The Third Affiliated Hospital of Sun Yat-Sen University, China.; ^3^Department of Neurology, Guangdong 999 Brain Hospital, Guangzhou, China.; ^4^Department of Cardiology, The First Affiliated Hospital of Sun Yat-Sen University, China.; ^5^Department of Neurology, Zhujiang Hospital, Southern Medical University, China.; ^6^School of Chinese Medicine, Hong Kong Baptist University, Hong Kong, China.; ^7^Department of Geriatric Medicine, Hunan Provincial People’s Hospital, Changsha, Hunan, China.; ^8^Department of Microbiology & Immunology, School of Medicine, New York Medical College, NY 10595, USA

**Keywords:** TFF3, ChE activity, Hcy, Parkinson disease dementia, vascular parkinsonism dementia, pathogenesis

## Abstract

Trefoil factor 3 (TFF3), cholinesterase activity (ChE activity) and homocysteine (Hcy) play critical roles in modulating recognition, learning and memory in neurodegenerative diseases, such as Parkinson’s disease dementia (PDD) and vascular parkinsonism with dementia (VPD). However, whether they can be used as reliable predictors to evaluate the severity and progression of PDD and VPD remains largely unknown. Methods: We performed a cross-sectional study that included 92 patients with PDD, 82 patients with VPD and 80 healthy controls. Serum levels of TFF3, ChE activity and Hcy were measured. Several scales were used to rate the severity of PDD and VPD. Receivers operating characteristic (ROC) curves were applied to map the diagnostic accuracy of PDD and VPD patients compared to healthy subjects. Results: Compared with healthy subjects, the serum levels of TFF3 and ChE activity were lower, while Hcy was higher in the PDD and VPD patients. These findings were especially prominent in male patients. The three biomarkers displayed differences between PDD and VPD sub-groups based on genders and UPDRS (III) scores’ distribution. Interestingly, these increased serum Hcy levels were significantly and inversely correlated with decreased TFF3/ChE activity levels. There were significant correlations between TFF3/ChE activity/Hcy levels and PDD/VPD severities, including motor dysfunction, declining cognition and mood/gastrointestinal symptoms. Additionally, ROC curves for the combination of TFF3, ChE activity and Hcy showed potential diagnostic value in discriminating PDD and VPD patients from healthy controls. Conclusions: Our findings suggest that serum TFF3, ChE activity and Hcy levels may underlie the pathophysiological mechanisms of PDD and VPD. As the race to find biomarkers or predictors for these diseases intensifies, a better understanding of the roles of TFF3, ChE activity and Hcy may yield insights into the pathogenesis of PDD and VPD.

Parkinson’s disease (PD) and vascular parkinsonism (VP) are among the most common neurodegenerative disorders. Longitudinal studies have shown that many PD patients will eventually develop into PD with dementia (PDD), leading to poorer activities of daily living and increased caregiver burden [1, 2]. Although patients with VP show different neuro-pathogenesis than patients with PD, they typically present with similar clinical characteristics as PD [3]. For instance, in the later stages, both diseases present severe non-motor dysfunction, including impaired cognition and depression [4-6]. Compared with patients with PD or healthy controls, VP patients display more profound cognitive declines with impaired attention [7]. However, risk factors and time to onset for PDD and VP dementia (VPD) vary from patient to patient, reflecting the inter-individual heterogeneity underlying the different pathological substrates of PDD and VPD [3, 8]. Although these risk factors of ongoing PDD and VPD have not been clearly defined, several clinical predictors have been suggested. One population-based cohort study indicated that low CSF levels of β-amyloid 42 (Aβ42) at PD diagnosis predict substantial increased risk of early progression to dementia in patients with incident PD [9]. Several lines of evidence have also indicated that uric acid (UA) is a major natural antioxidant that might have neuroprotective properties [10, 11]. Low serum UA levels have been associated with worse cognitive functioning later in life and also with higher risk and faster progression of PD and VP [11, 12].

**Table 1:** Demographic, motor, and non-motor parameters.

Clinical parameters		PDD			Healthy subjects			VPD		

		Mean (SD)	Min	Max	Mean (SD)	Min	Max	Mean (SD)	Min	Max
**Gender (n)**	Male n (%)	49(53.3)	/	/	44(55)	/	/	45(54.8)	/	/
	Female n (%)	43(46.7)	/	/	36(45)	/	/	37(45.2)	/	/
**Age (years)**		65.73(11.18)	56	88	64.43(7.10)	50	82	70.29(9.87)	65	83
**H&Y**		2.85(1.23)	1	5	/	/	/	2.68(1.05)	1	5
**MMSE**		21.72(3.94)	6	24	/	/	/	17.34(5.04)	1	23
**UPDRS**		50.92(23.13)	17	96	/	/	/	44.36(19.03)	16	83
	UPRDRS(I)	3.70(2.05)	1	12	/	/	/	3.79(3.08)	0	14
	UPRDRS(II)	18.17(9.64)	3	45	/	/	/	16.57(8.79)	3	40
	UPRDRS(III)	27.02(11.04)	9	51	/	/	/	22.49(9.58)	5	51
	UPRDRS(Ⅵ)	2.03(2.50)	0	9	/	/	/	1.52(1.81)	0	7
**NMSS (total)**		86.77(53.47)	20	188	/	/	/	103.22(44.42)	30	235
	Cardiovascular	4.06 (2.90)	0	12	/	/	/	4.96(3.95)	0	18
	Sleep/Fatigue	17.50(9.58)	0	36	/	/	/	19.64(8.90)	2	46
	Mood	19.30(14.41)	3	54	/	/	/	23.30(12.51)	0	56
	Perceptual problem	2.87(4.48)	0	18	/	/	/	3.47(5.92)	0	26
	Attention/memory	11.27(7.79)	0	30	/	/	/	14.51(7.11)	0	30
	Gastrointestina	9.35(6.54)	0	28	/	/	/	10.15(7.37)	0	31
	Urinar	8.30(8.16)	0	32	/	/	/	11.18(7.93)	0	36
	Sexual function	5.45(6.23)	0	18	/	/	/	8.41(7.69)	0	24
	Miscellaneous	8.67(10.25)	0	36	/	/	/	7.61(7.20)	0	38
**Daily dose of L-Dopa (mg)**		252.6(58.52)	206.44	291.14	/	/	/	275.06(65.77)	223.35	305.21
**Disease Duration**		4.05(3.40)	0.5	15	/	/	/	3.53(3.36)	0.5	11

SD, standard deviation; UPDRS, Unified Parkinson’s disease rating scale; H&Y, the modified Hoehn and Yahr staging scale; MMSE, mini-mental state examination and NMSS, non-motor symptoms scale for Parkinson’s disease.

Trefoil factor 3 (TFF3) is a neuropeptide secreted by secretory epithelial cells principally in the gastrointestinal tract and different cerebral regions including the hypothalamus, pituitary, hippocampi, temporal cortices and cerebellum [13-15]. Recent studies have shown that TFF3 may facilitate learning, objective recognition and retention of memory [16]. Low TFF3 in CSF is a predictive factor for brain atrophy and its potential role in the pathogenesis of Alzheimer disease (AD) has been suggested [17]. Multiple lines of evidence indicate that TFF3 is associated with vascular epithelial restitution [18] and anti-inflammatory effects and that it exerts protective effects against age-related diseases [17, 19]. Homocysteine (Hcy), a sulfur-containing amino acid produced by the interaction of cysteine and methionine, is closely related to cognitive impairment [20-23] and high levels can damage endothelial cells [24]. Although both TFF3 and Hcy are correlated with endothelial cell efficiency and declining cognition, the association of TFF3/Hcy levels with the severity of dementia in PD and VP has not been systemically evaluated.

Cholinesterase activity (ChE activity), including acetylcholinesterase (AChE) activity and butyryl-cholinesterase (BuChE) activity, has been studied as a marker for AD and plays a crucial role in preserving cognitive function [25, 26]. Previous studies have found that ChE activity levels reflect metabolic alterations associated with dementia, but they have not yielded information for their differential diagnoses [27, 28]. Several lines of evidence suggest that ChE/AChE activities contribute to regulating vascular endothelial dysfunction [29], suppressing inflammation and improving recovery prospects in cerebral ischemic diseases [30].

TFF3, Hcy and ChE activity modulate cognitive function and are associated with vascular function, inflammation and oxidative stress [17, 20, 24, 26, 29]. Vascular damage and chronic inflammation are observed in PDD and VPD patients [31-34]. Therefore, it is promising to investigate their levels and roles in PDD and VPD. The primary aim of this study was to compare serum levels of TFF3/ChE activity/Hcy among healthy subjects and patients with PDD and VPD. The secondary aim was to evaluate whether serum levels of TFF3/ChE activity/Hcy are associated with motor/non-motor dysfunctions in PDD and VPD. Lastly, we intended to determine the diagnostic value of serum levels of TFF3/ChE activity/Hcy in patients with PDD and VPD. Our study will provide a better understanding of the roles of TFF3, ChE activity and Hcy in the pathogenesis of PDD and VPD.

## MATERILAS AND METHODS

### Patients and Ethics Statement

This cross-sectional study was performed in the Department of Neurology of the Third Affiliated Hospital of Sun Yat-sen University, Guangzhou, P. R. China. From November 2013 to October 2016, a total of 174 dementia patients (PDD and VPD patients) were recruited into this study. A total of 92 PD patients with dementia (49 males and 43 females, Table 1) were enrolled in this study. Diagnosis of PDD patients was carried out consensually between 2 clinicians using the diagnostic criteria for PDD [35]. An additional 82 VPD patients with dementia (45 males and 37 females, Table 1) were recruited based upon the consensus criteria for the clinical diagnosis of VP. MMSE scores in all patients were less than 25. A total of 80 healthy subjects (44 males and 36 females, Table 1) were recruited from the outpatient setting as the control group. All the outpatients were recruited from the Medical Examination Centre in the Third Affiliated Hospital of Sun Yat-sen University. Exclusion criteria for the study included hypertension, cerebral ischemia, cardiovascular disease, diabetes, renal dysfunction diabetes or psychiatric diseases (e.g. depression, drug addiction). Patients were also excluded if they presented with abnormal prostate carcinoma-related mediators, including prostate-specific antigen (PSA), carcinoembryonic antigen (CEA) or alpha-fetoprotein (AFP).

This study was approved by the local Ethics Committee of the Third Affiliated Hospital of Sun Yat-sen University and was conducted in accordance with the principles outlined in the Declaration of Helsinki and the National Institutes of Health Human Subjects Policies and Guidance released on January and December 23, 1999, respectively. All participants provided written consent for the investigation and their consent to measure levels of serum TFF3, ChE activity and Hcy. Patients with PDD and VPD also completed additional disease-specific standard assessments, which were all conducted in a blinded manner.

### Study Design

Experienced neurologists were recruited to perform the evaluations and completed the neurological examinations for both the treatment and control participants. All patients with PDD included in this study satisfied the criteria presented in Emre’s reports [35]. PDD exclusion criteria were as follows: (1) PDD patients with disability due to neurological disorders other than PDD, such as cerebrovascular disease, sequelae or psychosis; (2) PDD patients with somatic diseases that could have a potential effect on NMS (e.g., pain syndromes, advanced diabetes mellitus, malignancy, renal, hepatic/heart failure, severe anemia, any other acute/chronic debilitating or life-threatening diseases/states); (3) MMSE scores higher than 25 (25 excluded); or (4) refusal to provide informed consent. All patients with VPD in this study fulfilled the criteria presented in Dunet and Zijlmans’ reports [31, 36] as follows: (1) parkinsonism, defined as bradykinesia and at least one of the following: resting tremor, rigidity or postural instability; (2) cerebrovascular disease, defined as evidence of relevant cerebrovascular disease by brain imaging or the presence of focal signs or symptoms that are consistent with stroke; (3) a relationship between these first two criteria and the following: an acute or delayed progressive onset of parkinsonism (within 1 year) after stroke with evidence of infarcts in or near areas that increase the basal ganglion motor output or decrease the thalamocortical drive directly OR an insidious onset of parkinsonism with extensive subcortical white matter lesions, bilateral symptoms at the onset and the presence of early shuffling gait or early cognitive dysfunction. All subjects completed the following battery of standard assessment measures: a standard demography form, the unified Parkinson’s disease rating scale (UPDRS) [37] and the modified Hoehn and Yahr staging scale (H&Y) [38]. The UPDRS(I) ‘mentation’ and UPDRS(II) ‘daily life’ subscales were used to evaluate psychiatric dysfunction and disease severity. The UPDRS (III) ‘motor’ and H&Y subscales were used to evaluate motor dysfunction and disease severity. The degree of non-motor symptoms (NMS) in every patient was measured by the NMS scale (NMSS) [5, 39, 40]. Cognitive abilities were evaluated with the Mini-Mental State Examination (MMSE) [5, 41]. All scales were available and validated for the Chinese population [5]. According to Reinoso and Oosterveld’s studies, we divided the PDD and VPD into three sub-groups with UPDRS-III scores respectively, namely mild (≤30 points, 28 cases for PDD, 21 cases for VPD), moderate ( 31-50 points, 39 cases for PDD, 36 cases for VPD) and severe ( >50 points, 25 cases for PDD, 25 cases for VPD) [42, 43]. All participants were scanned using magnetic resonance imaging (MRI) and representative MRIs for normal controls/PDD/VPD patients are shown in Figure 3. Demographic and clinical data for participants are shown in Table 1.

### Blood sampling measurement

Venous blood samples for TFF3, ChE activity and Hcy measurements were obtained from all subjects in the study. 5 ml of blood was drawn from the patients under fasting state in the morning and all of blood measurements were replicated thrice. The serum was separated for a total of 1?h by centrifugation at 3,000?rpm for 10?min. Separated sera were stored at -30°C until laboratory evaluation took place. Serum levels of TFF3 were measured using commercial ELISA kits purchased from R&D Systems (Minneapolis, MN, USA) and performed in accordance with the manufacturer’s instructions. ChE activity levels were measured using the method of Ellman et al. [44]. Serum levels of Hcy were determined using a solid-phase competitive chemiluminescent enzyme immunoassay [20].

**Table 2:** Comparison of age, MMSE, TFF3, Hcy and ChE activity among PDD, VPD and normal healthy subjects.

Variable	PDD	VPD	Control	*t* Value	*p* Value	Tukey’s		
						PDD/VPD	PDD/Control	VPD/Control
Age	65.73±11.18	70.29±9.87	64.43±7.10	2.925	0.008**	0.007**	0.295	0.005**
MMSE	21.72±3.94	17.34±5.04	30	3.523	0.001**	0.008**	0.000***	0.000***
TFF3	15.21±11.83	14.03±12.25	18.20±6.21	-2.668	0.006*	0.089	0.007**	0.005**
ChE activity	7528±1573	7232±1254	7785±1962	-1.815	0.042*	0.882	0.067	0.028*
Hcy	16.18±4.96	18.21±5.72	10.45±3.19	3.478	0.002**	0.625	0.008**	0.006**

* *p*<0.05,

** *p*<0.01,

*** *p*<0.001. Kruskal-Wallis test for the comparison among PDD, VPD and normal subjects, Tukey’s post hoc analysis for the comparison in PDD vs. VPD, PDD vs. Control, or VPD vs. Control.

### Statistical analyses

All continuous variables, including age, UPDRS (UPDRS-I, -II, -III and -IV), MMSE, NMSS, TFF3, ChE activity and Hcy, are presented as their means?±?SD. All categorical variables, including gender, are presented as percentages. Total scores for age, UPDRS, MMSE, NMSS, CRP, TFF3, ChE activity and Hcy were counted by summing the single items. The statistical significance of differences between the groups was assessed by Mann-Whitney U test and Kruskal-Wallis test when the data were not normally distributed; Student’s *t*-test was used when the data were normally distributed. One-way analysis of variance (One-way ANOVA) followed by Tukey’s *post-hoc* analysis was conducted to compare differences in TFF3/ChE activity/Hcy among normal subjects and PDD and VPD patients, including PDD and VPD sub-groups based on UPDRS(III) scores distribution. Spearman’s rank correlation coefficients (*r*_s_) were obtained to evaluate correlations among different clinical parameters. A receiver operating characteristic (ROC) analysis was conducted to assess the performance of clinical biomarkers (TFF3, ChE activity and Hcy) as diagnostic criteria for these diseases. Additionally, ROC curves for the combination of TFF3, ChE activity and Hcy were calculated as a possible better prognostic tool using logistic regression analysis. *P*-values <0.05 were deemed statistically significant and SPSS 13.0 (Chicago, IL, USA) was used for all statistical analyses.

**Table 3:** Comparison of TFF3, Hcy and ChE activity between normal subjects and PDD patients according to genders.

Variable		PDD (mean ± SD)	Control (mean ± SD)	PDD vs. Control		PDD(Male) *vs*. (Female)	

				Value	*p*	Value	*p*
TFF3	Male	15.03±11.26	20.12±9.51	-2.558	0.009**^b^	-2.976	0.003**^a^
	Female	17.29±10.17	20.43±7.10	-2.911	0.006**^a^		
ChE activity	Male	7596±1433	7736±1648	-2.083	0.038*^a^	-0.863	0.520^b^
	Female	7680±1259	7839±1542	-1.915	0.026*^b^		
Hcy	Male	18.21±4.72	11.39±2.80	3.974	0.000***^b^	3.239	0.003*^a^
	Female	14.59±5.73	10.74±2.25	3.015	0.002*^a^		

* *p*<0.05,

** *p*<0.01,

*** *p*<0.001.

a Mann-Whitney U-test.

b Student’s t-test.

## RESULTS

### Patient Characteristics

This cross-sectional study consisted of 92 PDD patients, 82 VPD patients and 80 healthy subjects (Table 1). The mean age of the VPD patients was higher than the mean age of the PDD patients (Table 2) and the mean age of the VPD patients was higher than the mean age of the normal subjects (Table 2). There were no significant differences in ages among the PDD patients and the healthy controls (Table 2). Demographic and clinical data for all subjects are shown in Table 1.

### Comparisons of Age, MMSE, TFF3, ChE activity and Hcy between PDD/VPD Patients and Healthy Subjects

In this study, the mean age for patients with VPD was higher than that of the PDD patients (Table 2) and the MMSE for patients with VPD was lower than that of PDD patients (Table 2). Significant differences in serum levels of TFF3/ChE activity/Hcy were found among the PDD and VPD patients and the control subjects (Table 2). Serum levels of TFF3 in PDD/VPD patients were lower than in normal subjects (Table 2), while no significant differences in TFF3 levels between PDD and VPD patients were observed. Similarly, ChE activity levels in patients with VPD were significantly lower than in the healthy subjects (Table 2). Serum Hcy levels in PDD/VPD patients were higher than in the normal subjects (Table 2), but no significant differences in Hcy levels between PDD and VPD patients were found.

When PDD and VPD patients and normal subjects were divided into specific gender groups, the serum levels of TFF3 in the male/female patients with PDD were lower than those of the normal male/female subjects (Table 3). Similarly, a significant decrease was observed in plasma ChE activity between PDD male/female patients and normal male/female subjects (Table 3). However, a significant difference was observed in plasma Hcy levels between PDD male/female patients and normal male/female subjects (Table 3). Additionally, in PDD patients, levels of TFF3 in male patients were lower than in female patients (Table 3), while serum Hcy levels in male patients were higher than those in female patients with PDD (Table 3). There were no significant differences in serum ChE activity levels between male PDD patients and female PDD patients (Table 3). Interestingly, our data showed that serum TFF3 and ChE activity levels in male/female VPD patients were significantly lower than in male/female normal subjects (Table 4), while Hcy serum levels were significantly higher than in male/female normal subjects (Table 4). TFF3 levels in male VPD patients were lower than in the female VPD patients (Table 4), while serum Hcy levels in male VPD patients were higher than those in female VPD patients (Table 4). There were no significant differences in the serum ChE activity levels between male VPD patients and female VPD patients.

**Table 4:** Comparison of TFF3, Hcy and ChE activity between healthy subjects and VPD patients according to genders.

Variable		VPD (mean ± SD)	Control (mean ± SD)	VPD vs. Control		VPD(Male) *vs*. (Female)	

				Value	*p*	Value	*p*
TFF3	Male	14.67±11.51	20.12±9.51	-3.956	0.000*^a^	-3.241	0.004**^a^
	Female	16.98±12.36	20.43±7.10	-3.579	0.002*^b^		
ChE activity	Male	7286±1626	7736±1648	-2.066	0.032*^b^	-0.571	0.739^b^
	Female	7351±1820	7839±1542	-2.017	0.041*^a^		
Hcy	Male	21.11±5.24	11.39±2.80	3.923	0.000***^a^	2.939	0.003**^b^
	Female	16.27±4.19	10.74±2.25	3.381	0.000***^b^		

* *p*<0.05,

** *p*<0.01,

*** *p*<0.001.

a Mann-Whitney U-test.

b Student’s t-test.

### Comparison of TFF3, Hcy and ChE activity between PDD and VPD patients according to genders and UPDRS-III scores

We found serum Hcy levels in male VPD patients were significantly higher than those in male PDD patients (Table 5), while serum TFF3 and ChE activity didn’t display such gender-associated differences between PDD and VPD patients (Table 5). When PDD and VPD groups were divided into three sub-groups based on UPDRS-III scores respectively, we found serum TFF3 levels were significantly lower in VPD patients than those in PDD patients in UPDRS(III)≤30 and 31<UPDRS(III)<50 sub-groups. Besides, our data showed that serum ChE activity were significantly lower in VPD patients than those in PDD patients in 31<UPDRS(III)<50 and UPDRS(III)>50 sub-groups (Table 5).

### Correlation analyses among TFF3/ChE activity and Hcy Levels in PDD and VPD Patients

**Figure 1.**
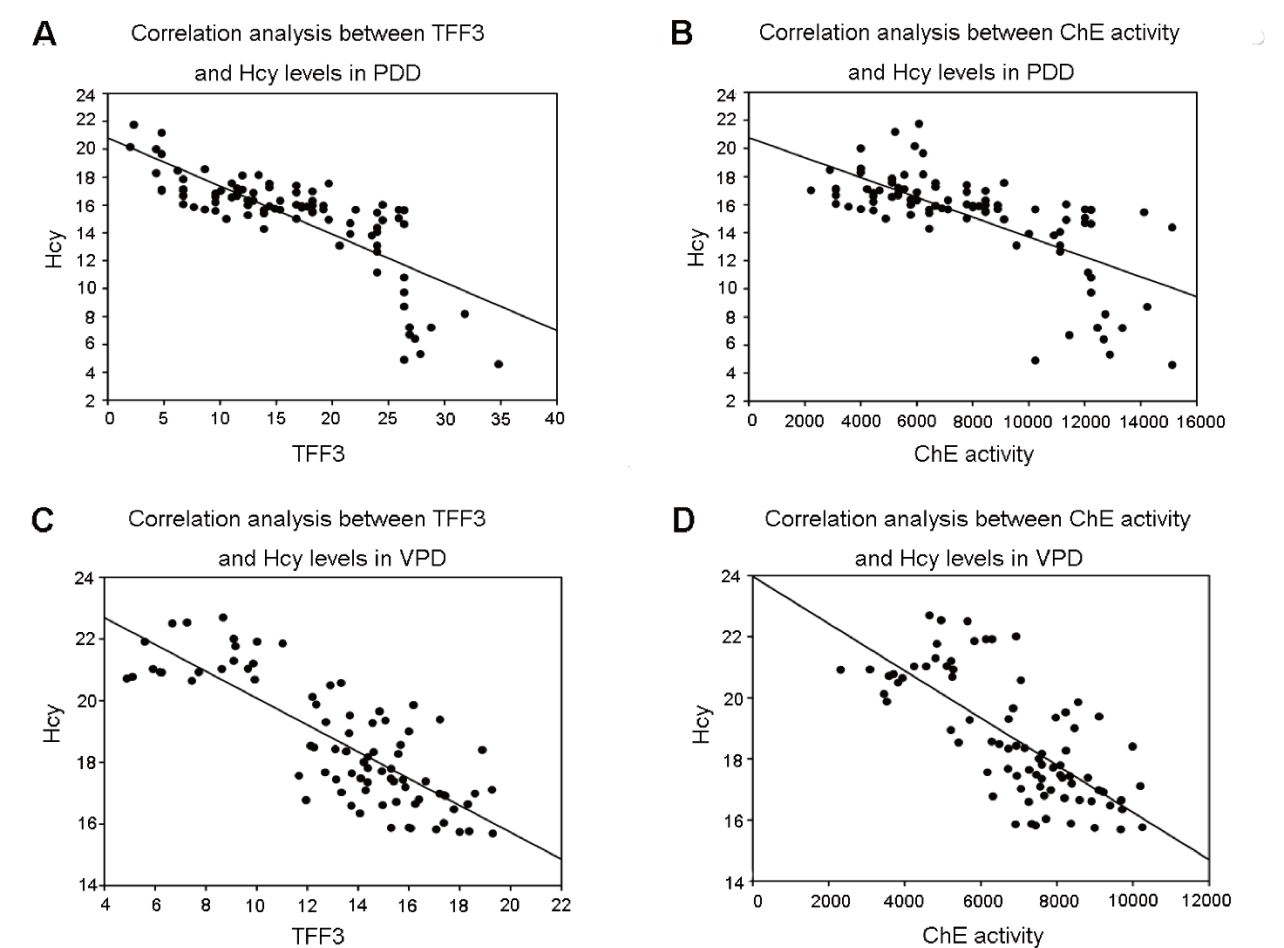
Correlation analysis between TFF3/ChE activity and Hcy Levels in PDD and VPD patients A significant negative correlation between (**A**) TFF3 and Hcy Levels in PDD patients (*r_s_* =-0.799, ***p<0.001); (**B**) ChE activity and Hcy Levels in PDD patients (*r_s_* =-0.732, ***p<0.001). (**C**) TFF3 and Hcy Levels in VPD patients (*r_s_* =-0.771, ***p<0.001). (**D**) ChE activity and Hcy Levels in VPD patients (*r_s_* =-0.713, ***p<0.001).

To evaluate the relationships between TFF3/ChE activity and Hcy Levels in PDD and VPD patients, we conducted Spearman’s correlation analysis. We found a significant negative correlation between TFF3 and Hcy Levels in PDD and VPD patients (Table 6, Fig. 1). Similarly, a profound inverse correlation between ChE activity and Hcy levels was observed in PDD and VPD patients (Table 6, Fig. 1).

### Correlations among TFF3/ChE activity/Hcy levels and age, UPDRS, H&Y, MMSE and NMSS (total/domain) in PDD and VPD Patients

To evaluate correlations between the severity of the disease and clinical variables in PDD and VPD patients, we conducted Spearman’s correlation analysis among mediator variables and various assessments. We found TFF3 level was negatively correlated with UPDRS, UPDRS(Ⅲ) and H&Y staging and positively correlated with MMSE scores in PDD and VPD patients (Table 7). Although no significant correlations were observed for TFF3 and UPDRS(I), TFF3 and UPDRS(II), TFF3 and UPDRS(IV) or TFF3 and some of burdens of NMSS, there were significant correlations between TFF3 and NMS burdens of mood, attention/memory, as well as gastrointestinal distress in PDD and VPD patients (Table 7).

Besides, serum Hcy was positively correlated with H&Y staging and negatively correlated with MMSE scores in PDD and VPD patients (Table 7). Specifically, positive correlations were observed between Hcy and NMSS-cardiovascular burden in PDD and VPD patients, and between Hcy and NMSS-attention/memory burden in VPD patients (Table 7). There were, however, no significant correlations between Hcy and UPDRS, Hcy and UPDRS(I), Hcy and UPDRS(II), Hcy and UPDRS(Ⅲ), Hcy and UPDRS(IV) or Hcy and NMSS in either PDD/VPD patients.

Additionally, we found serum ChE activity was negatively correlated with UPDRS, UPDRS(Ⅲ), H&Y staging and positively correlated with MMSE scores in PDD and VPD patients (Table 7). There were negative correlations between ChE activity and the NMSS-cardiovascular burden in VPD patients, and between ChE activity and NMS burdens of mood and attention/memory in PDD and VPD patients (Table 7). No significant correlations were observed between ChE activity and UPDRS (I/II/IV), ChE activity and NMSS in PDD and VPD patients. Interestingly, our results also showed that, although patients with PDD and VPD were treated with L-dopa, this L-dopa treatment was not significantly correlated to their serum levels of TFF3, Hcy or ChE activity (Table 7). This finding strongly suggests that L-dopa medication in PDD and VPD patients does not influence the validity of TFF3/Hcy/ChE activity assessment in those PDD and VPD patients.

#### ROC Curves for TFF3, ChE activity and Hcy in the Diagnosis of PDD and VPD

**Figure 2.**
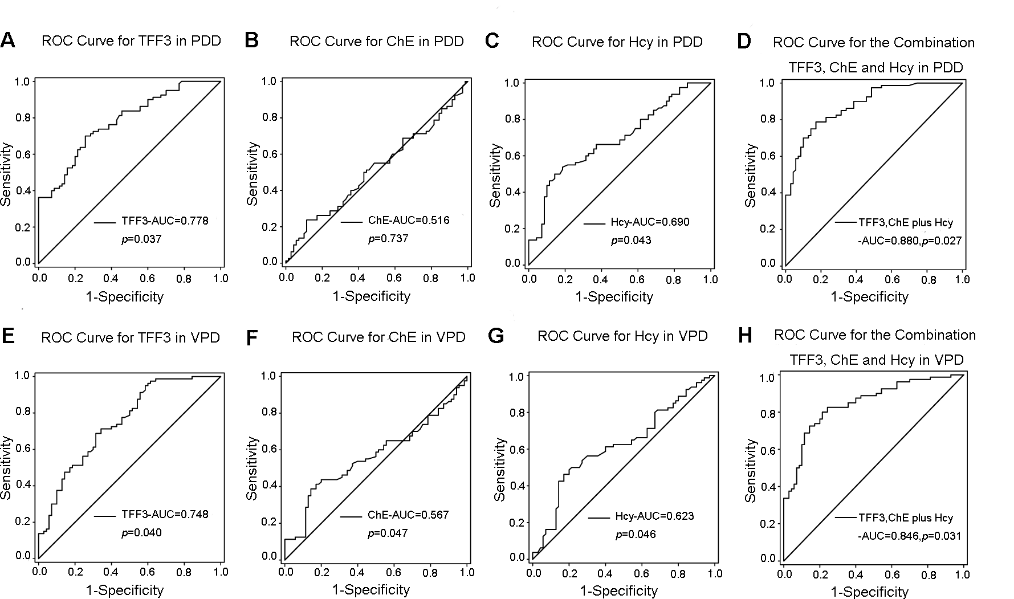
ROC curves to evaluate the utility of serum levels of TFF3, ChE activity and Hcy Levels for the discrimination of PDD/VPD patients from healthy controls (**A-D**) The AUC of ROC curves for discrimination of PDD patients from healthy controls (**A**) TFF3, (**B**) ChE activity, and (**C**) Hcy were 0.778 (95%CI: 0.706-0.850, *p=0.037), 0.516 (95%CI: 0.423-0.609, p=0.737), and 0.690 (95%CI: 0.606-0.774, *p=0.043), respectively. The AUC of (**D**) TFF3+ChE activity+Hcy was 0.880 (95%CI: 0.828-0.932, *p=0.027). (**E-H**) The AUC of ROC curves for discrimination of VPD patients from healthy controls. (**E**) TFF3, (**F**) ChE activity, and (**G**) Hcy were 0.748 (95%CI: 0.671-0.826, *p=0.040), 0.567 (95%CI: 0.475-0.660, *p=0.047), and 0.623 (95%CI: 0.533-0.713, *p=0.046), respectively. The AUC of (**H**) TFF3+ChE activity+Hcy was 0.846 (95%CI:0.785-0.908, *p=0.031).

ROC curves were constructed to explore whether TFF3, ChE activity and Hcy levels could provide credible discrimination between PDD patients and normal subjects. ROC curves for TFF3 analysis revealed that an area under the curve (AUC) value of 0.778 (Fig. 2A) was appropriate; the cut off was at 15.39 μmol/L, with a sensitivity of 62% and specificity of 90%. The AUC for Hcy was 0.690 (Fig. 2C); the cut off was at 16.82 μmol/L, with a sensitivity of 54% and specificity of 86%. However, the AUC for ChE activity was 0.516 (Fig. 2B), indicating no significant differences. The AUC for the combination of TFF3, Hcy and ChE activity was 0.880 (Fig. 2D), with a sensitivity of 68% and a specificity of 89% at a cutoff of 0.59 on the predicted risk algorithm.

**Table 5:** Comparison of TFF3, Hcy and ChE activity between PDD and VPD patients according to genders and UPDRS-III scores.

Variable		PDD Mean ± SD	VPD Mean ± SD	PDD vs. VPD	
				Value	*p*
TFF3					
Gender	Male	15.03±11.26	14.67±11.51	0.153	0.8786
	Female	17.29±10.17	16.98±12.36	0.902	0.123
UPDRS(III)	UPDRS(III)≤30	16.85±10.33	15.37±10.16	2.629	0.011^a^*
	31<UPDRS(III)<50	15.74±10.29	14.16±10.03	3.140	0.003 a**
	UPDRS(III)≤30	14.33±9.81	13.85±9.27	1.218	0.082
ChE activity					
Gender	Male	7596±1433	7286±1626	0.982	0.3285
	Female	7680±1259	7351±1820	0.951	0.345
UPDRS(III)	UPDRS(III)≤30	8027±1003	7829±1579	1.883	0.075
	31<UPDRS(III)<50	7829±1282	7320±1050	2.145	0.037 a*
	UPDRS(III)>50	7462±1291	7057±1331	2.335	0.020 a*
Hcy					
Gender	Male	18.21±4.72	21.11±5.24	2.832	0.006^b^**
	Female	14.59±5.73	16.27±4.19	1.476	0.144
UPDRS(III)	UPDRS(III)≤30	17.82±3.94	19.33±4.91	0.232	0.713
	31<UPDRS(III)<50	16.35±4.31	18.56±4.43	0.537	0.281
	UPDRS(III)>50	15.78±4.60	17.41±3.89	0.891	0.371

* *p*<0.05,

** *p*<0.01,

*** *p*<0.001.

a Tukey’s post hoc analysis.

b Mann-Whitney U-test.

Furthermore, in VPD patients, the AUC for TFF3 was 0.748 (Fig. 2E); the cut-off was at 14.21 µmol/L, with a sensitivity of 60% and a specificity of 75%. The AUC for Hcy was 0.623 (Fig. 2G); the cut off was at 18.36 μmol/L, with a sensitivity of 48% and specificity of 66%. However, the AUC for ChE activity was 0.567 (Fig. 2F); the cut off was at 7250 U/L, with a sensitivity of 44% and specificity of 56%. The AUC for the combination of TFF3, Hcy and ChE activity was 0.846 (Fig. 2H), with a sensitivity of 57% and a specificity of 76% at a cutoff of 0.53 on the predicted risk algorithm. These data indicate that the combination variable was more robust than TFF3, ChE activity or Hcy alone in distinguishing PDD and VPD patients from healthy controls.

## DISCUSSION

We found several important results in this study. First, we found a pronounced decrease in the serum levels of TFF3 and ChE activity and an increase in the levels of Hcy in PDD/VPD patients when compared to healthy subjects. Second, increased serum Hcy levels were significantly and inversely correlated with decreased TFF3/ChE activity levels. Third, there were significant correlations between TFF3/ChE/Hcy levels and the severity of PDD/VPD, including motor dysfunction, declining cognition and mood/gastrointestinal symptoms. Lastly, our ROC curve analysis strongly indicated that the combination of TFF3, ChE activity and Hcy could significantly discriminate PDD/VPD patients from healthy subjects and could be applied as a potential screening instrument for disease diagnosis. To our knowledge, this is the first study to explore changes in the serum levels of TFF3 and ChE activity in PDD/VPD patients and to evaluate the potential relationships between TFF3 and the dementia. The interesting finding of inversed correlation between serum TFF3/ChE activity and Hcy levels may shed light on the underlying pathogenesis of PDD and VPD.

**Figure 3.**
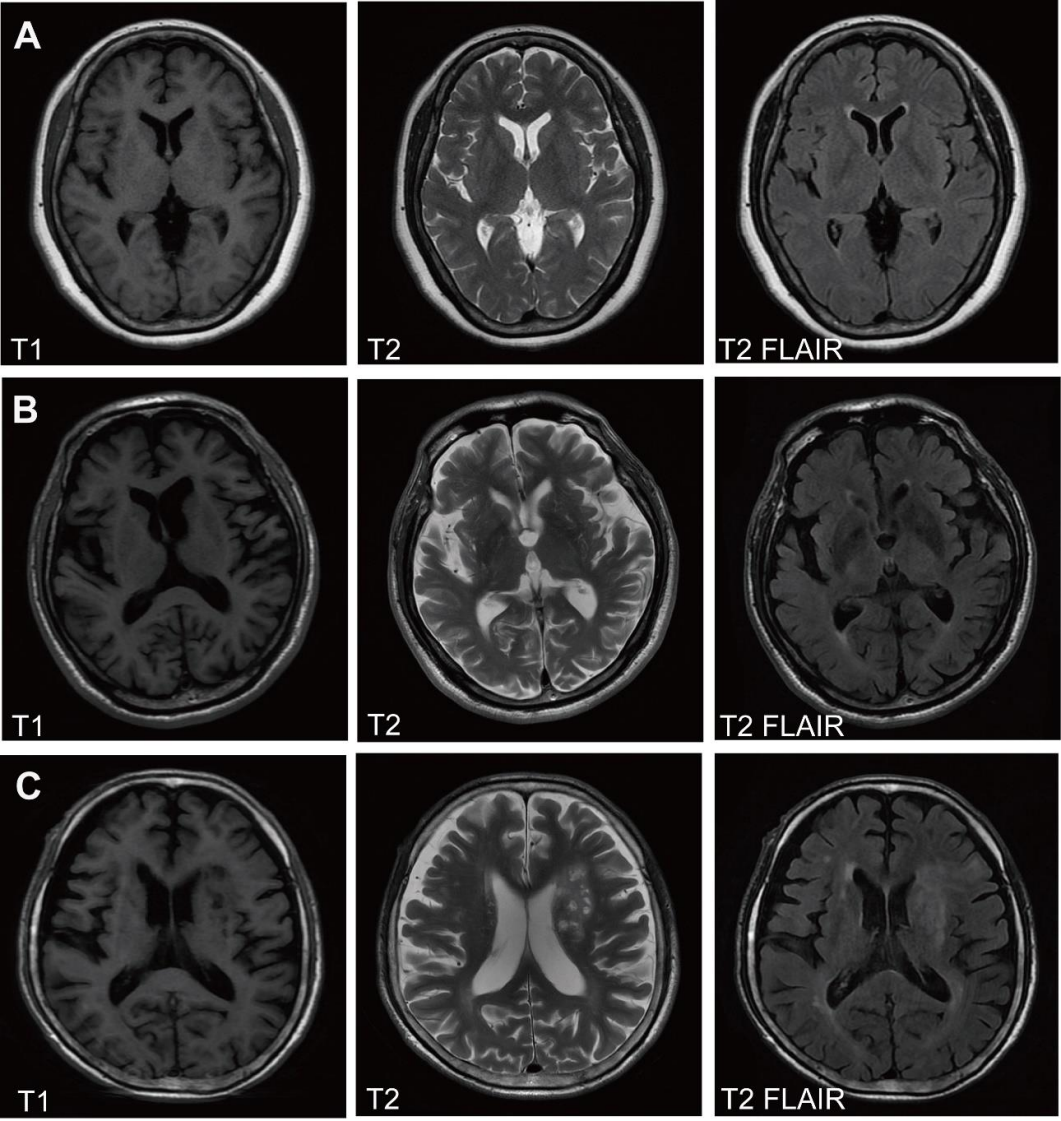
MRI images in normal controls and PDD and VPD patients (**A**) Normal subjects, (**B**) PDD patients, (**C**) VPD patients. The extent of white matter hyperintensities and multiple infarctions in the basal ganglia in the VPD patients are shown in T2-weighted and FLAIR images. Arrows indicate the infarction.

**Table 6:** Spearman’s rank correlation coefficient (rs) and *p*-values between TFF3/ChE activity and Hcy Levels in PDD and VPD patients.

Variable	Hcy in PDD		Hcy in VPD	

	*r*	*p*	*r*	*p*
TFF3	-0.799	0.000***	-0.771	0.000***
ChE activity	-0.732	0.000***	-0.713	0.000***

* *p*<0.05,

** *p*<0.01,

*** *p*<0.001. *r*_s_, Spearman’s rank correlation coefficient.

Over the past few years, comparisons of VPD and PDD patients have received significant attention for the diagnosis, therapy and evaluation of their status [36, 45-48]. We found that NMS (including cardiovascular, sleep and mood disorders) are very common in patients with PDD and VPD, with a prevalence of NMS in the group of 100% and an NMSS for PDD (Table 1) consistent with previous reports [39, 49]. Interestingly, as shown in Tables 1 and 2, our patients with VPD exhibited shorter disease durations and older ages at onset than those patients with PDD. This finding implies that Chinese VPD patients may develop symptoms later in life, but they may experience deterioration faster than PDD patients. Additionally, a later age at onset of VP would favor a vascular cause [31]. Significant reductions in scores on the MMSE were observed in VPD patients when compared to the PDD group (Table 2). This result demonstrates that compared to PDD subjects, Chinese VPD patients had already undergone a greater decline in cognitive function by the time they visited the neurologist. This result is in agreement with Zijlmans’ finding [36] and implies that our VPD patients may have more subcortical lesions than the patients with PDD and these subcortical vascular ischemic lesions may have led to their more rapidly declining cognition [7].

Until now, the roles of TFF3 in the central nervous system have not been adequately explored. TFF3 has been previously documented to be associated with cognitive functioning, including vascular epithelial restitution and suppressing inflammation in the central nervous system [16-19]. TFF3 levels in patients with PDD/VPD were significantly lower than in the healthy subjects, similar to findings from other neurodegenerative diseases such as AD [17]. When compared to healthy controls, these decreased TFF3 levels in PDD/VPD patients imply that TFF3 contributes to the pathophysiological mechanisms of disease development. Serum ChE activity reflects metabolic alterations associated with dementia [50]. Szilagyi et al. described lowered serum and CSF ChE activity in patients with dementia [51]. We also found that serum ChE activity in VPD patients was significantly lower than that in healthy subjects. Although serum ChE activity in PDD patients was lower than in healthy subjects, the difference did not reach significance. One possible explanation may be that these declines in ChE activity in the CNS are not low enough to affect peripheral concentrations in PDD patients [52]. It has been well documented that altered TFF3 and ChE activity are associated with a higher risk of AD. Low CSF TFF3 levels are associated with both the rate of cognitive decline and the rate of hippocampal atrophy and ventricular expansion [17]. Several lines of evidence have indicated that significantly lower cortical and thalamic AChE activity are associated with declines in cognitive impairment [52]. Therefore, in the diagnosis and evaluation of PDD and VPD, TFF3 and ChE activity could be used as potential markers.

Previous studies have demonstrated that elevated plasma Hcy levels represent a risk factor for declining cognition and dementia in the general population [22, 53], but Hcy levels have also been associated with mild cognitive impairment (MCI), AD, PD, and vascular dementia [20, 23, 54, 55]. Similar results have been observed in PDD and VPD patients. These findings strongly suggest that higher levels of Hcy may contribute to the severity of PDD and VPD. Therefore, Hcy may be a possible target in the treatment of PDD and VPD [54, 56].

After dividing subjects by gender, we noticed (Table 4) that serum TFF3 levels in male PDD/VPD patients were significantly lower than female PDD/VPD patients, while serum Hcy levels in male PDD/VPD patients were significantly higher than in female PDD/VPD patients. This finding suggests that serum TFF3 and Hcy may be more useful in evaluating PDD/VPD in men. These bio-physiological associations between gender-specific hormones and TFF3/Hcy may be partly attributable to higher occurrences of PDD/VPD in men [57, 58].

Interestingly, we observed no significant difference in serum TFF3, ChE activity and Hcy levels between PDD and VPD patients. This finding may be due to the similar neuro-pathogenesis between PDD and VPD, *i.e*. those three factors participating the vascular epithelial restitution and suppressing inflammation in the brain [18, 19, 22, 26, 29]. We next explored the differences between PDD and VPD patients. Although the three serum biomarkers displayed no difference between these two groups, serum Hcy levels were higher in male VPD patients than those in male PDD patients (Table 5), implying Hcy may be more suitable to evaluate the status and more closely correlated to the neuro-pathogenesis in male VPD patients [58]. When PDD and VPD groups were divided into three sub-groups based on UPDRS-III scores respectively, we found serum TFF3 levels were significantly lower in VPD patients than those in PDD patients in UPDRS(III)≤30 and 31<UPDRS(III)<50 sub-groups. This finding suggests that in VPD patients, especially in the early-mid stage, micro-vascular impairment and inflammatory response are more prominent, as indicated by the lower levels of serum TFF3 in VPD compared to PDD [4, 6, 18, 19]. Interestingly, we noticed that serum ChE activity was significantly lower in VPD patients than those in PDD patients in 31<UPDRS(III)<50 and UPDRS(III)>50 sub-groups (Table 5). This result strongly implies that during mid-later stage, cerebral function is more impaired and more neurons are damaged in VPD compared to PDD, as shown by lower ChE activity in VPD [25, 26]. Whether serum TFF3 and ChE activity could be used in evaluating the status of VPD and PDD needs further exploration.

**Table 7:** Spearman’s rank correlation coefficient (*r*_s_) and *p*-values between clinical variables and H&Y, MMSE, NMSS(total/domain) s in PDD and VPD patients.

Variable	TFF3 (PDD)		Hcy (PDD)		ChE activity (PDD)		TFF3 (VPD)		Hcy (VPD)		ChE activity (VPD)	

	*r*	*p*	*r*	*p*	*r*	*p*	*r*	*p*	*r*	*p*	*r*	*p*
Age	0.423	0.091	0.138	0.550	0.168	0.433	0.474	0.087	0.257	0.375	0.134	0.647
UPDRS	-0.126**	0.007	0.134	0.053	-0.319*	0.019	-0.795**	0.001	0.193	0.195	-0.367*	0.015
Up(Ⅰ)	-0.096	0.521	0.169	0.256	-0.270	0.237	-0.133	0.535	0.030	0.220	-0.463	0.096
Up(Ⅱ)	-0.091	0.541	0.173	0.247	-0.134	0.367	-0.458	0.065	0.143	0.289	-0.197	0.355
Up(Ⅲ)	-0.327*	0.025	0.283	0.213	-0.397*	0.015	-0.578*	0.015	0.073	0.274	-0.298*	0.017
Up(Ⅳ)	-0.052	0.645	0.042	0.712	-0.037	0.742	-0.266	0.106	0.185	0.079	-0.037	0.745
H&Y	-0.315*	0.031	0.342*	0.019	-0.269*	0.025	-0.206*	0.047	0.306*	0.026	-0.379*	0.022
MMSE	0.378**	0.009	-0.364*	0.012	0.358*	0.014	0.249*	0.026	-0.339*	0.030	0.418**	0.008
NMSS	0.241	0.103	0.006	0.978	0.065	0.681	0.086	0.074	0.127	0.071	0.049	0.462
Cardiovascular	-0.205	0.168	0.359*	0.015	-0.201	0.178	-0.094	0.138	0.115*	0.014	-0.207*	0.048
Sleep/Fatigue	-0.478	0.084	0.417	0.064	-0.057	0.755	-0.026	0.616	0.085	0.218	-0.041	0.526
Mood	-0.351*	0.015	0.067	0.681	-0.391**	0.001	-0.343*	0.028	0.070	0.601	-0.393**	0.001
Perceptual problem	-0.058	0.218	0.080	0.253	-0.185	0.105	-0.074	0.646	0.054	0.392	-0.035	0.831
Attention/ memory	-0.118*	0.014	0.009	0.950	-0.249*	0.026	-0.243*	0.029	0.362*	0.013	-0.341*	0.034
Gastrointestina	-0.668**	0.009	-0.141	0.341	-0.069	0.645	-0.769**	0.001	0.113	0.103	-0.112	0.075
Urinar	-0.045	0.763	0.018	0.915	-0.021	0.894	-0.063	0.190	0.054	0.462	-0.205	0.198
Sexual function	-0.219	0.140	0.029	0.846	-0.227	0.164	-0.083	0.071	0.073	0.301	-0.250	0.115
Miscellaneous	-0.044	0.350	-0.007	0.919	-0.208	0.155	-0.410	0.081	0.230	0.119	-0.104	0.487
Daily dose of L-Dopa (mg)	-0.071	0.130	0.131	0.058	-0.100	0.497	-0.031	0.502	0.027	0.695	-0.002	0.972

*p<0.05,

** *p*<0.01,

*** *p*<0.001. *r*_s_, Spearman’s rank correlation coefficient; UPDRS, Unified Parkinson’s disease rating scale; H&Y, the modified Hoehn and Yahr staging scale; MMSE, mini-mental state examination; NMSS, non-motor symptoms scale for Parkinson’s disease.

TFF3 and ChE activity showed strong negative correlations with UPDRS, H&Y and NMSS (mood and attention/memory) and positive correlations with MMSE in PDD/VPD patients. This finding strongly suggests that serum levels of TFF3 and ChE activity may significantly influence motor and non-motor dysfunction in PDD and VPD patients. Previous studies have indicated that TFF3 deficiency presents with a significant forelimb motor dysfunction in experimental ischemic injury mice [59]. Significant correlations were also observed between decreased cortical AChE activity and higher total UPDRS scores in patients with PD [52, 60]. These findings suggested that the ascending cholinergic system from the nucleus basalis of Meynert to the cerebral cortex is impaired more severely as PD advances. We also found similar findings for ChE activity, with significant correlations between lower ChE activity and higher UPDRS/UPDRS III scores in PDD and VPD patients. Specifically, TFF3 and ChE activity showed negative associations with mood and attention/memory and positive correlations with MMSE in PDD/VPD patients, further demonstrating that these two dysfunctions could be important targets for disease evaluation using plasma levels of TFF3 and ChE activity. Consistent with our notions, several studies have shown that TFF3 deficiency and low levels of ChE activity are correlated with mood, memory deterioration and cognitive function [16, 50]. We also noticed plasma levels of TFF3 showed robust associations with the gastrointestinal domain in PDD and VPD patients, implying that TFF3 could be a high-risk factor for gastrointestinal disease. Interestingly, Hcy demonstrated strong positive correlations with H&Y and NMSS (cardiovascular domain) and a negative correlation with MMSE in PDD/VPD patients, implying that plasma Hcy levels might be used to evaluate the severity of cognitive status of PDD and VPD patients [61, 62].

Since several lines of evidence show that TFF3, ChE and Hcy may be involved in the pathogenesis of dementia [16, 22, 23, 26], we specifically explored the relationships among serum TFF3, ChE and Hcy. In the current study, we noticed a significant and inverse correlation between Hcy and TFF3/ChE activity in PDD and VPD patients (Table 6, Figure 1). Previous studies have indicated that elevated plasma Hcy increases the risk of dementia by impacting cerebrovascular pathology [5, 6, 63, 64]. These higher levels of Hcy in PDD/VPD patients may damage neuron and vascular endothelial cells in the brain, inducing neuroinflammation [23, 65, 66]. High levels of Hcy could break down the blood brain barrier (BBB) and lead to cerebrovascular dysfunction, which subsequently modulates the activities of enzymes and neuropeptides and influences hippocampal volume in the pathophysiological processes of dementia [67-69]. It has been shown that Hcy could stimulate superoxide and hydrogen peroxide generation in vivo and in vitro [70] and that ChEs activities are inhibited by high level of Hcy mediated by the generation of free radical formation [71-73]. Multiple lines of evidence indicate that TFF3 exerts protective effects via promoting vascular epithelial restitution and anti-inflammation,which is opposite to the effects of Hcy [17-19]. Therefore, we propose that Hcy may negatively influence cognitive functioning in PDD/VPD patients via altering cerebrovascular pathology and subsequently downregulating TFF3/ChE activity, subsequently leading to impaired cognition in PDD and VPD. Based on this finding, we hypothesize that the combination of TFF3, ChE activity and Hcy would be an early pathophysiological marker for cognitive dysfunction in VP and PD patients.

Our ROC data indicate an acceptable sensitivity and specificity for TFF3 and ChE activity in the potential discrimination of PDD/VPD patients from normal subjects. Notably, TFF3 displays more reliable discrimination when compared to ChE activity (Figures 2A, D). Moreover, one of the most notable findings in the current study showed that the combination of TFF3 and ChE activity and Hcy exhibited better discriminatory ability in PDD/VPD, in comparison to TFF3, ChE activity or Hcy alone. With the use of the serum TFF3 and ChE activity as screening tools, clinicians could potential use these as a combined biomarker to potentially detect PDD/VPD.

There are several limitations to this study: (1) a small number of participants (92 PDD patients, 82 VPD patients, and 80 healthy subjects) were recruited. Therefore, it is necessary to conduct a large population study in the future; (2) most patients with PDD/VPD were at middle-late stages of the disease, with a high median stage on the H&Y scale and a relatively low MMSE score (21.72 for PDD patients, 17.34 for VPD patients); (3) our study is a cross-sectional study. Therefore, longitudinal cohort studies are needed in the future to explore the alterations of the three serum biomarkers (TFF3, ChE and Hcy) during the disease progression in PDD and VPD; (4) genetic factors such as the *TFF3* genotype and folate or cholinesterase inhibitor administration were not considered in this study; (5) to validate and complete the questionnaire, we chose only PDD and VPD patients with sufficient cognitive ability, which significantly narrowed the study population. The above criteria (1-5) in the population chosen may have resulted in a bias for serum levels of TFF3, ChE activity and Hcy in PDD/VPD patients. Therefore, it is necessary to conduct a large population study in the future.

In summary, the current study supports the notion that decreased serum TFF3/ChE activity and increased Hcy may be related to the pathophysiology of PDD and VPD. Based on this study, it is reasonable to speculate that high levels of serum Hcy may negatively influence cognitive function in PDD/VPD patients through various mechanisms, including inducing cerebrovascular pathology and subsequent downregulation of TFF3/ChE activity. Furthermore, low levels of serum TFF3 and ChE activity combined with high levels of serum Hcy may predispose patients to the progressive stages in both motor and non-motor dysfunctions. Based on our findings, we propose that serum TFF3, ChE activity and Hcy may underlie the pathophysiological mechanisms of PDD/VPD and could be used to evaluate the severity of these diseases.
